# Intimate partner violence and associated factors among reproductive age women during COVID-19 pandemic in Southern Ethiopia, 2020

**DOI:** 10.1186/s12978-021-01297-3

**Published:** 2021-12-13

**Authors:** Solomon Shitu, Alex Yeshaneh, Haimanot Abebe

**Affiliations:** 1 Department of Midwifery, Wolkite University College of Health and Medical Sciences, Wolkite University, PO BOX 07, Wolkite, Ethiopia; 2 Department of Public Health, Wolkite University College of Health and Medical Sciences, Wolkite University, Wolkite, Ethiopia

**Keywords:** Intimate partner violence, COVID**-**19, Reproductive age, Gurage, Ethiopia

## Abstract

**Background:**

World health organization (WHO) defines intimate partner violence (IPV) is physical, sexual, or emotional abuse by an intimate partner or ex-partner or spouse to a woman. From all forms of violence, ~ 1.3 million people worldwide die each year, accounting for 2.5% of global mortality. During the COVID-19 crisis, control and prevention measures have brought women and potential perpetrators together which increase the risk of IPV. Therefore, this study was aimed to assess the magnitude and associated factors of IPV against women during COVID-19 in Ethiopia.

**Methods:**

Community based cross-section study was employed among 462 reproductive-age women to assess IPV and associated factors during COVID-19 pandemic. To select study participants one-stage cluster sampling technique was used. The data were entered into Epi data version 4.2 and exported to SPSS for analysis. Bivariate and multivariate analysis was used to check the association of dependent and independent variables and statistical significance was declared at P < 0.05.

**Result:**

A total of 448 study subjects were responded making a response rate of 96.97%. Two- third (67.6%) of the respondent's age range was between 20 and 29 years. All of the participants heard about the pandemic of COVID-19 at the time of onset. The lifetime and the last twelve months prevalence of women with IPV was 42.19% and 24.11%, respectively. About 58 (12.9%) had experienced all three types of violence. Participants age ≥ 35 (AOR = 2.02; 95% CI: 1.99–4.29), rural residence (AOR = 3.04; 95% CI: 2.59–6.25), husband’s educational status of diploma and above (AOR = 0.35; 95% CI: 0.14–0.83), COVID-19 pandemic (AOR = 4.79; 95% CI: 1.13–6.86), and low social support (AOR = 3.23; 95% CI: 1.99–6.23) were independent predictors.

**Conclusions:**

In this study two in five women undergo one type of violence in their lifetime. The occurrence of the COVID-19 pandemic has its impact on violence. Age ≥ 35, rural residence, husband’s educational status of diploma and above, history of child death, COVID-19 pandemic, and low social support were independent predictors of violence. This implies insight to concerned bodies like policymakers and stakeholders to design appropriate policies to avert this magnitude and making zero tolerance for violence in society.

**Supplementary Information:**

The online version contains supplementary material available at 10.1186/s12978-021-01297-3.

## Plain English summary

Intimate partner violence is physical, sexual, or emotional abuse by an intimate partner or ex-partner or spouse to a woman. Community based cross-section study was employed among 462 reproductive-age women to assess the magnitude and associated factors of IPV against women during COVID-19 in Ethiopia. Bivariate and multivariate analysis was used to check the association of dependent and independent variables and statistical significance was declared at P < 0.05. A total of 448 study subjects were responded making a response rate of 96.97%. In this study two in five women undergo one type of violence in their lifetime. The occurrence of the COVID-19 pandemic has its impact on violence. Age ≥ 35, rural residence, husband’s educational status of diploma and above, history of child death, COVID-19 pandemic and low social support were independent predictors of violence. This sparks light to concerned bodies like policymakers and stack holders to design appropriate policies to avert this magnitude and making zero tolerance for violence in society.

**Supplementary Information:**

The online version contains supplementary material available at 10.1186/s12978-021-01297-3.

## Introduction

Intimate partner violence (IPV) is physical, sexual, or emotional abuse by an intimate partner or ex-partner or spouse to a woman as described by the WHO [1]. It is renowned worldwide as a substantial public health problem and against the right of women. About 35% of women worldwide have experienced either physical or sexual violence by an intimate male partner/cohabiting partner, parents, or any family relative [2]. The lifetime prevalence of sexual, physical, or both sexual and physical violence among women ranges from 15 to 71% [3].

Intimate partner violence has been pronounced as a real violation of women's rights before the start of the Corona Virus Disease in 2019 (COVID-19) pandemic [4]. However, remarkably in Africa and across the globe, it has increased during the COVID-19 crisis, control and prevention measures targeted at reducing COVID-19 have exacerbated the situation of already vulnerable women. Isolations and stay-home protective measures and movement restrictions related to COVID-19 have brought women and potential perpetrators together under the confines of the home setting which have been seen to increase the risk of IPV [5–7].

Current lockdown declaration in several areas of the developed countries like UK, USA, France, and Alberta call reports have increased by 20%, 21–35%, 32–36%, and 30–50%, respectively [6]. Besides, in China, Italy, and Spain, IPV call emergency has been increasing during the COVID-19 pandemic [8]. Available data in Africa showed that 36.6% of women experienced lifetime physical and/or sexual IPV among ever partnered women [9]. During the COVID-19 pandemic, majorly in Kenya, Somalia, South Africa, Niger, Tunisia, Zimbabwe, and Ethiopia, IPV has been reported as high as before [10]. In specific Addis Ababa Ethiopia, within less than two months, during the era of COVID-19, more than 100 girls have been raped, some of them by close family members [11].

From all forms of violence, ~ 1.3 million people worldwide die each year, accounting for 2.5% of global mortality [12]. For women who survive from IPV, it has many adverse consequences including pregnancy and its complications, cognitive and behavioral problems, mental health problems and poor emotional well-being, behaviors presenting risks to health including alcohol and drug misuse and eating disorders, and physical injuries are some of them [3, 13–15].

Many global pieces of evidence on the increasing domestic violence cases are probable to continue throughout the pandemic and may only show a “tip of the iceberg” as many victims still find themselves trapped with the perpetrator and unable to report the abuse [16]. By considering the aforementioned problems, the voice of the violated women as a result of the COVID-19 pandemic needs to be heard and some sort of urgent actions need to be taken to avoid one woman dying every three days due to IPV related to COVID-19 quarantine [4]. Therefore, this study aimed to assess the magnitude and associated factors of intimate partner violence which offer vital information for policymakers, program planners, and other stakeholders who have interests to stop violence against women in Ethiopia.

## Methods

### Study design area and period

A community-based quantitative cross-sectional was conducted from September 20 to October 10/2020 in Gurage Zone, Southern Ethiopia. The Zone is one of the zones of southern nation’s nationalities and peoples of Ethiopia region (SNNPR). It is located 256 km away from Hawassa the capital city of the region and 158 km southwest of Addis Ababa the capital city of Ethiopia. The zone is composed of sixteen districts and five town administrations. According to the 2007 national household census, the Gurage zone has a total population of 1,279,646, of which 657,568 are women and 622,078 are men [17].

### Population

All reproductive age women living in the Gurage zone were source populations. While those randomly selected reproductive-age women living in the Gurage zone were study populations.

### Eligibility criteria

All reproductive age women living in the study area and/or reside at least six months before data collection and ever live with partner were included. Those participants who were seriously ill and unable to respond during the data collection period were excluded.

### Sample size determination and sampling procedure

The sample size was determined by a computer-based Epi info 7 software Stat Cal using single population proportion formula with the assumption of 95% confidence interval, P = Intimate partner violence against reproductive age women during COVID-19 pandemic in northern Ethiopia 2020 (p = 24.6%)[18], d = is a tolerable margin of error (d = 0.05) and 10% non-response rate, we used design effect of 1.5 because the sampling procedure was one-stage cluster sampling, then the calculated final sample size for the study is 462.

A one-stage cluster sampling method was used to draw the final sample size. The zone has sixteen districts and five town administrations. From those districts and town administrations, we selected four districts (Cheha, Hawariat, Ener and Geta districts) and two town administrations (Emdeber and Butajira) by a simple random sampling technique (lottery method). Finally, a systematic random sampling technique was used to include participants from selected districts and town administrations by using kth value calculated for the proportion of the population living in each districts and town administrations. The list of reproductive age women was obtained from health extension workers and we used it as sampling frame.

### Method of data collection

The information was collected by face-to-face interview and it was prepared by reviewing different kinds of literature. In Ethiopia, the Amharic version of the questionnaire has been validated as a screening tool in Addis Ababa with sensitivity and specificity of 79.9 and 78.3, respectively. The questionnaire included socio-demographic factors of both the participant and her partner, obstetrics-related factors, decision-making power, alcohol use, the behavior of the husband [18–21, 23]. Wealth index was computed using principal component analysis [23]. To assess the outcome variable validated and structured questionnaire was adapted from WHO core questions to assess domestic partner violence [2]. The outcome was measured by 13 questions (three questions for sexual violence, four questions for emotional violence, and six questions for physical violence) if partner committed one of these 13 questions he violated his wives. Social support was measured by using the scale containing six questions scoring 0–5 Likert scales each and adopted from Maternity Social Support Scale. The score was categorized into three high, medium, and low social supports with a value of score 24–30, score 18–23, and below 18, respectively [24]. The English language questionnaire prepared first and then was translated to the local language *Amharic* (the language spoken in the area). Ten BSc holder health professionals were recruited for data collection and they were supervised by two MSc holder health workers. The principal investigators overseas the whole activity and coordinate the process (Additional file 1).

### Data quality assurance

Two days of training were given for data collectors and supervisors about the process of data collection briefly including the tool. The questionnaire was first prepared in the English language and was translated to local language for clarity and back-translated to the English language then comparison was made on the consistency of the two versions. The pretest was done to assess the validity of the tool and possible amendment and clarity were done based on the result before the actual data collection. The supervisors were checked on the field and reviewed the questionnaires to ensure completeness and consistency of collected data. The interview was done in private with adequate confidentiality by making interview away from somebody can listen what she responded to minimize bias. Double data entry was done to see the consistency of the entered data and cross-checked by comparing the two separately entered data.

### Dependent variables

Intimate partner violence.

### Independent variables

Socio-demographic and socio-economic factors of both the participant and her partner, obstetrics related factors, COVID-19 related issues, decision making power of the women, controlling the behavior of the partner, alcohol use, behavior of the husband, social support, number of children, history of child death.

### Operational definition

#### Physical violence

The husband or co-habitant acting one of the six acts (something thrown to harm her, pushed, hit with a fist or something else to hurt her, kicked, drugged, burnt on purpose, perpetrator try to use or used gun, knife against her) [25]

#### Sexual violence

The husband or co-habitant acting one of the following three acts (forced sexual intercourse against her interest, sexual intercourse when she didn't want to due to afraid of what he might do and she forced to do something sexual that she perceives as humiliating) [25].

#### Emotional violence

If the participant says yes for one of the following. Her partner was insulted or made her to feel bad about one self, humiliated her in front of others, intimidated or scared her on purpose, or threatened her with harm [26].

#### Lifetime IPV

The women reported the experience of having one or more acts of physical or sexual violence by a current or former partner at any point in time [25, 26].

#### Twelve months violence

The women reported physical or sexual violence by their partner in the last twelve months period [25, 26].

#### Controlling behavior

If the women report that her partner acts one of the following four behaviors (restricts her from seeing her friends, contact with her family, insists on knowing where she is all the time, and gets angry when she speaks with other men [25].

#### Social support

Social support of the women was classified into three categories based on the score; High social support (for scores 24–30), Medium social support (18–23), Low social support (below 18) [24].

### Data processing and analysis

The data were coded cleaned edited and entered into Epi data version 4.2 to avoid logical errors and then exported to SPSS version 24 for analysis. The analysis was done by computing proportions and summary statistics. Then the information was presented by using texts, tables, and figures. Bi-variate and multivariate analyses were computed to see the association between independent and dependent variables by using binary logistic regression. All variables with P ≤ 0.25 [18] in the bivariate analysis were included in the final model to control all possible confounders. Multi-collinearity was checked to see the linear correlation among the independent variables by using standard error. Variables with a standard error of > 2 were dropped from the multivariable analysis. Model fitness was checked with the Hosmer–Lemeshow test. The direction and strength of statistical association were measured by the adjusted odds ratio with 95% CI. The adjusted odds ratio along with 95% CI was estimated to identify factors by using multivariate analysis in the binary logistic regression. In this study P-value < 0.05 was considered to declare a result as statistically significant.

## Result

### Socio-demographic characteristics of the respondents

A total of 448 study subjects were responded making a response rate of 96.97%. Two- third (67.6%) of the respondent's age range was between 20 and 29 years and the age distribution ranged from the smallest 17 to the largest 46 years with a mean of 26.5 (SD ± 4.07) years. More than two-thirds of participant’s 309 (69.1%) was rural residents. One-fourth 122 (27.2%) of study subjects completed secondary education while 148 (37.9%) of the respondent’s husbands were diploma and above educational level. Among respondents, 390 (87.1%) participants were currently married and the majority of 193 (43.1%) women's occupation was a housewife. Of the respondents 236 (52.6%) were Orthodox religion followers while 33 (7.4%) were protestant. Two hundred fifty-five (56.9%) of participant's wealth index was in the second quintile (Table 1).

**Table 1:** Sociodemographic characteristics of women on the study has done IPV and associated factors, Southern Ethiopia, 2020

Variable	Classification	Frequency	Percentage (%)
Age	< 19	22	4.9
	20–24	159	35.5
	25–29	144	32.1
	30–34	82	18.3
	≥ 35	41	9.1
Marital status	Married	390	87.1
	Divorced/widowed	21	4.6
	Single	37	8.3
Residence	Rural	309	69
	Urban	139	31
Religion	Orthodox	236	52.6
	Muslim	120	26.8
	Catholic	59	13.3
	Protestant	33	7.3
Occupational status	Housewife	193	43.1
	Merchant	42	9.4
	Government employer	56	12.5
	Farmer	87	19.4
	Daily laborer	18	4
	Student	52	11.6
Educational status	No formal education	96	21.5
	Completed Primary school	105	23.4
	Completed secondary school	122	27.2
	Diploma and above	125	27.9
Occupation of the husband (n = 390)	Merchant	108	27.7
	Farmer	134	34.4
	Government employer	96	24.6
	Daily laborer	52	13.3
Husband’s educational status (= 390)	No formal education	66	16.9
	Completed Primary school	80	20.5
	Completed secondary school	116	29.8
	Diploma and above	128	32.8
Wealth index	Third quintile	121	27
	Second quintile	255	56.9
	First quintile	72	16.1

### COVID-19 related conditions

All of the participants heard about the COVID-19 pandemic at the time of onset. Only 72 (16.1%) were tested for COVID-19 once and the result was negative while the rest of them were never tested. About 136 (30.4%) of respondents have at least one family member tested for COVID-19. Three hundred seventy (82.6) were stayed lockdown at home at the time of the national state of the emergency announcement but know they are doing their work by applying safety precautions. From safety precautions 101 (22.6%) apply physical distancing, wear a mask, and use sanitizer, 165 (36.8%) only wear a mask, 88(19.6%) apply physical distancing, 94 (21%) use sanitizer after touching something.

### Social support and decision making power

**Fig. 1:**
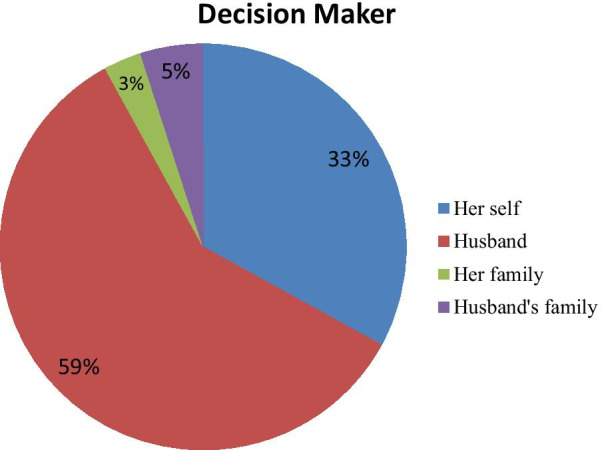
Decision making power of study participants in the study done to assess IPV and associated factors in Southern, Ethiopia, 2020

Almost half 221 (49.3) of the respondent’s social support was medium followed by low and high 131 (29.2%), 96 (21.4%), respectively. The participants who are ever married, the marriage were arranged for 264 (64.2%) by one of their family while the others were married by love each other. Of those respondents, 148 (33%) have decision-making power on their day-to-day activity while the others were guided by another second party (Fig. 1).

### Obstetric characteristics

From the study participants more than half 249 (55.6%) were gravid two and three but, 141 (31.5%) were nulliparous. From those who had a previous history of pregnancy nearly three fourth 290 (78.1%) had ANC follow-up at least once in their pregnancy and 61 (16.4%) had a history of abortion. Thirty (8.01%) of participants had a previous history of child death. Of those who had a history of birth 264 (85.9%) gave the last birth at a health institution. From total participants who had a history of delivery 58 (18.9%) had similar sex of their babies while the rest were mixed-sex (Table 2).

**Table 2:** Obstetric characteristics of the participants who participated in the study done on IPV in Southern Ethiopia, 2020

Variable	Classification	Frequency	Percentage (%)	
Gravidity	≤ One	141		31.5
	Two–three	249		55.6
	Greater three	58		12.9
History of abortion (n = 372)	Yes	61		16.4
	No	311		83.6
ANC in current/previous (n = 372)	Yes	290		78.1
	No	82		21.9
History of child death (n = 307)	Yes	30		8.01
	No	277		91.99
Last baby place of delivery (n = 307)	Hospital	161		52.5
	Health center	103		33.5
	Home	43		14
Sex of index baby (n = 307)	Male	167		54.4
	Female	140		45.6
Babies sex (n = 307)	Similar	58		18.9
	Mixed	249		81.1
Child illness (n = 307)	Yes	66		21.5
	No	241		78.5
No of alive baby (n = 307)	1	54		17.6
	≥ 2	253		82.4

### Controlling behavior husband and alcohol use

**Fig. 2:**
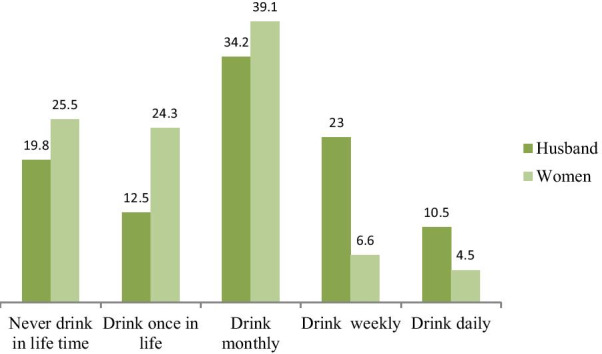
History of alcohol use of the study participants and their husbands in the study done to assess IPV and associated factors in Southern Ethiopia, 2020

Three-fourth 334 (74.5%) of respondent’s drunk alcohol at least once in their lifetime while the rest had never drunk alcohol. From the total of 390, respondent husbands 113 (28.9%) drink alcohol once a week while 41 (10.5%) drink alcohol daily (Fig. 2). The participants respond that 221 (56.7%) of respondent’s partners showed at least one sign of controlling behavior in their relation time.

### Women’s attitude towards violence

The study participant’s entire attitude towards physical violence was negative and culturally not acceptable. But 141 (31.47%) of the study participants said that emotional violence is no harm to them. While 91 (20.3%) of study subjects respond that sexual violence at home after marriage is normal and sometimes it can be the right of her husband to get pleasure by sexual intercourse with his wife without her interest.

### Prevalence of intimate partner violence

**Fig. 3:**
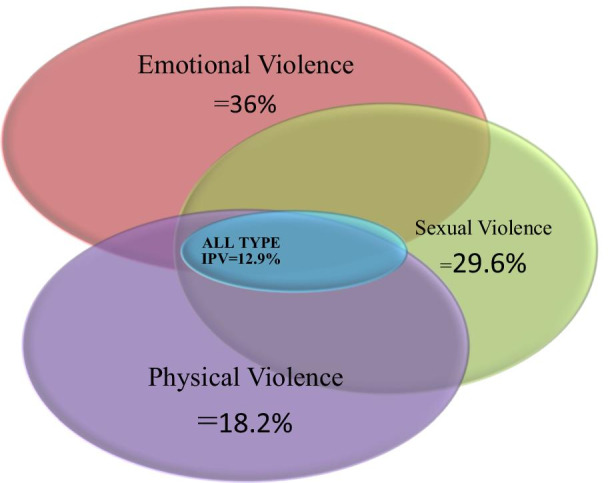
Venn diagram indicating overlaps overall, physical, emotional, and sexual violence in the study done to assess IPV and its predictors in Southern Ethiopia, 2020

In this study, the lifetime and the last twelve months prevalence of women with IPV were 189 (42.19%) with 95% CI: (39.3–45.9) and 24.11 with 95% CI: (20.23–29.32), respectively. From this 18.2%, 36%, and 29.6% of them reported physical, emotional, and sexual violence at least once in their relationship time, respectively. About 58 (12.9%) of the study participants had experienced all three types of violence (Fig. 3).

### Predictors of intimate partner violence

The covariates of this study were: age, residence, husbands educational status, decision making power, social support, wealth index, history of abortion, arranged marriage, history of child death, controlling behavior of the husband, COVID-19 pandemic, and similar sex of the children was a candidate for the multivariable model. In the multivariable model age, residence, husband’s educational status, social support, COVID-19 pandemic, and history of child death were statistically significant with intimate partner violence.

Participants age ≥ 35 were 2.42 (AOR = 2.42; 95% CI: 2.19–4.77) times more likely to undergo IPV than age group ≤ 19. Rural residents were 2.77 times (AOR = 2.77; 95% CI: 2.12–7.33) more likely to experience IPV than their counterparts. Husband’s educational status of Diploma and above were 0.65 (AOR = 0.24; 95% CI: 0.14–0.79) times less likely to commit IPV than those husbands who had no formal education. Participants who had a previous history of child death were 2.68 (AOR = 2.68; 95% CI: 1.48–4.77) times more likely to experience at least one type of IPV. Intimate partner violence has 4.21 (AOR = 4.21; 95% CI: 2.73–7.42) times more likely to occur after the COVID-19 pandemic than before the pandemic. Participants who had low social support were 3.66 times more likely to undergo IPV than those who had high social support (AOR = 3.66; 95% CI: 2.08–5.13) (Table 3).

**Table 3:** Factors associated with IPV in the study done to assess IPV and associated factors among reproductive-age women during covid-19 in Southern Ethiopia, 2020

Variables	IPV		(95% CI)	
	Yes	No	Crude OR	Adjusted OR
Age				
≤ 19	6 (3.2)	16 (6.2)	1	1
20–24	42 (22.2)	117 (45.2)	0.96(0.44–1.99)	1.66 (0.78–4.21)
25–29	62(32.8)	82(31.6)	2.02(1.08–4.23)	0.88 (0.26–3.54)
30–34	46(24.3)	36(13.9)	3.41(2.59–4.59)	1.23 (0.66–3.12)
≥ 35	33(17.5)	8(3.1)	11(9.02–13.65)	2.42 (2.19–4.77)*
Residence				
Urban	47 (24.9)	92 (35.5)	1	1
Rural	142 (75.1)	167 (64.5)	1.66(1.21–2.04)	2.77 (2.12–7.33)*
Husbands educational status *(n* = *390)*				
No formal education	32(22.2)	34(13.8)	1	1
Completed 1^ry^ school	29(20.1)	51(20.7)	0.60(0.41–1.77)	0.32 (0.55–6.25)
Completed 2^ry^ school	39(27.1)	77(31.3)	0.53(0.37–1.11)	0.56 (0.16–2.35)
Diploma and above	44(30.6)	84(34.2)	0.55(0.19–0.88)	0.24 (0.14–0.79)**
Timing of occurrence of IPV				
Before covid-19	121 (61.2)	179 (69.1)	1	1
After covid-19	68 (38.8)	80 (30.9)	3.98 (2.32 – 9.21)	4.21 (2.73–7.42)***
Children death (n = 307)				
Yes	22 (22.4)	8 (3.8)	7.27 (3.84—13.61)	2.68 (1.48–4.77)****
No	76 (77.6)	201 (96.2)	1	1
History of abortion (n = 372)				
Yes	40(24.5)	21(10)	2.91(1.02–5.21)	1.02 (0.87–4.33)
No	123(75.5)	188(90)	1	1
Social support				
High	33 (17.5)	63 (24.3)	1	1
Medium	74 (39.1)	147 (56.7)	0.96(0.66–1.79)	1.23 (0.76–1.36)
Low	82 (43.4)	49 (18.9)	3.19(2.18–4.21)	3.66 (2.08–5.13)****
Decision-making power				
Her self	96 (50.8)	52 (20.1)	1	1
Others	93(49.2)	207 (79.9)	0.24(0.33–0.89)	0.99(0.79–3.26)
Sex of their children (n = 307)				
Similar	46	12	4.46(3.75–5.21)	1.81(0.26–3.63)
Mixed	115	134	1	1

Others = husband, her family, husbands family

^*^Significant at P ≤ 0.002, **Significant at P ≤ 0.001, ***Significant at P ≤ 0.000, ****Significant at P ≤ 0.003

## Discussion

In this study, the prevalence of IPV was 42.19% with 95% CI 39.3–45.9. The finding indicated that two in five women were exposed to one form of IPV in the study area. The increase in the prevalence justifies there is limited intervention by different stakeholders and policymakers about the problem in the society. The finding was in line with the studies done in the Tigray region (40.8%), Debre Markos town (41.1%), and Harari region (39.8%) [21, 22, 27]. But the finding was lower than the studies done in Southeast Oromia (64.6%), Dilla town (51%), Wolita Zone (59.7%), and Ambo district (77%) [28–31]. And the finding was higher than the studies done in Tanzania, Shire Endasellassie town, and Northern Ethiopia [13, 18, 19]. The reason may be due to the difference in the study area, period including the occurrence of the pandemic, and cultural difference. Some of the studies were done only on urban residents or focused on specific characteristics of the population [21].

This study determined that the last year IPV was 24.11% this finding was in line with the study done in northern Ethiopia (24.6%) [18]. The average increase in the last year may be due to the stay at home or lockdown policy because of the COVID-19 pandemic. It increases the condition due to long time stay at home together that may lead to violence. Also, the lockdown affects all aspects of the population including socio-economic conditions that leading to conflict with each other and violence.

Age group ≥ 35 were 2.24 times more likely to undergo IPV than age group ≤ 19. The finding was supported by the studies done in East Wollega zone, 2016 EDHS, Debre Markos, and Wolita [21, 30, 32, 33]. The reason may be since age group < 19 were less likely to have sexual exposure as compared to the age group > 35 so this increase in exposure may increase exposure to violence and as the age advances the time of marriage also increases so partner may commit violence on one occasion due to different reasons. Also, this might be the prevalence is a cumulative report of the respondents throughout their exposure.

The study indicates that rural residents were 2.77 times more likely to experience IPV than their counterparts. This can be because rural residents are of low socioeconomic and educational status. Also since there is a lack of information about civilization and equality so that the women herself perceives and accept violence as normal. The finding was supported by studies done in different regions of Ethiopia [13, 21, 33]. But the study done in East Wollega Zone contradicts this finding [32].

Husband’s educational status of diploma and above were 24% less likely commit IPV than those husbands who had no formal education. The finding was in line with the studies done in Shire Endselassie, Dilla town, Debre Markos town, and Ambo district [13, 21, 29, 31]. This may be due to the fact that education changes the mindset of the people in different aspects. And also those who are educated people's way of communication is better than their counterparts. So, they perceive problems can be solved by dialog than violating the right of others.

Participants who had a previous history of child death were 2.68 times more likely to experience at least one type of IPV. The finding was similar to the study has done in Bale Zone [20]. The reason may be due to those husbands who had grave history related to child loss had most likely disappointed by the reason that happened so they may violate the right of his wife.

Those who had low social support were 3.66 times more likely to undergo IPV than those who had high social support. This can be justified as those who had low social support are less likely to be supported by her family this can lead to negligence and carelessness of her husband end up committing violence. Those who have low social support were mostly low economic status and rural area so there may not have a guarantee that keeps them from violation by husbands or co-habitant. The finding was supported by studies done in Northern Tanzania and Dilla town [19, 29].

Intimate partner violence has 4.21 times more likely to occur after the COVID-19 pandemic than before the pandemic. This indicates that how much COVID-19 has an impact on violence. The reason might be during the COVID-19 pandemic there is a vicious circle that is locked down and stay at home, keeping them at the home long period leading to a low socio-economic condition that predisposes them to conflict and violence. The finding is in line with the study done during the pandemic in northern Ethiopia [18].

## Conclusion

Two in five women undergo one type of violence in this study area. The occurrence of the COVID-19 pandemic has its impact on violence. Violated women as a result of the pandemic needs to be heard and urgent actions need to be taken to avoid one woman dying every three days due to IPV related to COVID-19 quarantine [4]. Age ≥ 35, rural residence, husband's educational status of diploma and above, history of child death, covid-19 pandemic, and low social support were independent predictors of violence. It needs cooperation and integration of different stakeholders to avert this violence in the society. And this study alarms the concerned bodies to work on preventing and educating the community about intimate partner violence because all forms of IPV have great impact on women’s health throughout the rest of her life.

### Strength

The study was done during the pandemic of covid-19 to find the factors associated with the outcome of interest. As the knowledge of investigators, this study was the first at specific study area so it will be used as a baseline for other studies.

### Limitation

Information was obtained by asking the respondents, so this may incur recall bias. And due to cultural sensitivity, it was susceptible to social desirability bias women might report socially acceptable responses than their actual day-to-day living that may underestimate the prevalence of the outcome.

Due to cross-sectional study, it is difficult to distinguish the cause and effect relationship.

## Supplementary Information

**Additional file 1.** This is the questionnaire to assess the Intimate Partner Violence and Associated Factors among Reproductive Age Women during Covid-19 Pandemic in Southern Ethiopia, 2020.

## Data Availability

The datasets used and/or analyzed during the current study available from the corresponding author on reasonable request.

## Abbreviations

ANC: Ante-Natal Care
COVID-19: Corona Virus Disease in 2019
IPV: Intimate Partner Violence
EDHS: Ethiopia Demographic Health Survey
WHO: World Health Organization
SD: Standard Deviation
SDG: Sustainable Development Goal
SNNPR: South Nation Nationalities Peoples Republic
SPSS: Statistical Package for Social Sciences
WKU: Wolkite University
