# Supplementary material for: Intimate partner violence and associated factors among reproductive age women during COVID-19 pandemic in Southern Ethiopia, 2020
Source: Reprod Health. 2021 Dec 13;18:246. doi: 10.1186/s12978-021-01297-3 (PMC8667533; doi:10.1186/s12978-021-01297-3)
Supplement: Supplementary file 1 — Additional file 1. This is the questionnaire to assess the Intimate Partner Violence and Associated Factors among Reproductive Age Women during Covid-19 Pandemic in Southern Ethiopia, 2020. [file 12978_2021_1297_MOESM1_ESM.docx]

**This is the questionnaire to assess the Intimate Partner Violence and Associated Factors among Reproductive Age Women during Covid-19 Pandemic in Southern Ethiopia, 2020**

01. Keble: ________________

02. House code__________________

03. Code of the Women__________________

03. Name of Data Collector: ____________ Signature__________ Date: _______

04. Name of Supervisor: ___________ ______ Signature_______ Date: _____**__**

| \|  \| \| --- \|  \|  \| \| \| \| \| \| \| \| \| \| \| \| --- \| --- \| --- \| --- \| --- \| --- \| --- \| --- \| --- \| --- \| --- \| \| **S NO** \| \| **Question** \| \| \| **Response** \| \| \| \| **Skip** \| \| \| **PART I: SOCIO-DEMOGRAPHIC CHARACTERISTICS** \| \| \| \| \| \| \| \| \| \| \| \| 101 \| Age of the mother \| \| /_______/ in complete year \| \| \| \| \| \|  \| \| \| 102 \| Marital status \| \| 1. Married  2. Single  3. Divorced  4. Widowed \| \| \| \| \| \|  \| \| \| 103 \| Religion \| \| 1. Orthodox  2. Protestant  3. Catholic  4. Muslim  5. Traditional  6. If other specify................ \| \| \| \| \| \|  \| \| \| 104 \| Educational status \| \| 1. Cannot able to read and write  2. Can read and write  3. Grade 1-8  4. Grade 9-12  5. diploma and above \| \| \| \| \| \|  \| \| \| 105 \| Occupation \| \| 1. House wife  2. Merchant  3. Government employer  4. Farmer  5. Daily laborer  6. Student  7. Other, Specify_______ \| \| \| \| \| \|  \| \| \| 106 \| Educational status of Husband \| \| 1. Cannot able to read and write  2. Can read and write  3. Grade 1-8  4. Grade 9-12  5. diploma and above \| \| \| \| \| \|  \| \| \| 107 \| Husband’s occupational \| \| 1. Farmer  2. Merchant  3. Student  4. Civil servant  5. Day laborer  6. Others specify \| \| \| \| \| \|  \| \| \| **PART II: SOCIO- ECONOMIC STATUS/ WEALTH INDEX QUESTIONS** \| \| \| \| \| \| \| \| \| \| \| \| 201 \| Does any member of this household has own any agricultural land? \| \| 1. Yes  2. No \| \| \| \| \| \| If “No” skip to Q202 \| \| \| 202 \| How much (local units) of agricultural land does members of this household own? \| \| _____________( in Hectare) \| \| \| \| \| \|  \| \| \| 203 \| Is the land cultivated? \| \| 1. Yes 2. No \| \| \| \| \| \| If “No” skip to Q204 \| \| \| 204 \| How much land is cultivated \| \| _____(in Hectare) \| \| \| \| \| \|  \| \| \| 205 \| Does your family have any stored grains/cereals in the house? \| \| 1. Yes  2. No \| \| \| \| \| \| If “No” skip to Q206 \| \| \| 206 \| How many “kunital”? \| \| _______(in number) \| \| \| \| \| \|  \| \| \| 207 \| Does this household own any livestock, herds, other farm animals, or poultry? \| \| 1. Yes  2. No \| \| \| \| \| \| If “No” skip to Q208 \| \| \| 208 \| How many of the following animals, does this household own? \| \| \| Animals \| No \| \| --- \| --- \| \| Milk cows, oxen or bulls \|  \| \| Horses, donkey or mules \|  \| \| Goet \|  \| \| Sheep \|  \| \| Chicken \|  \| \| Beehives \|  \| \| \| \| \| \| \|  \| \| \| 209 \| Which of the following does your household have? *(Record observation,Multiple response is possible)* \| \| 1. Electricity 2. Radio  3. Television 4. Mobile Telephone  5. Non Mobile Telephone  6. Chair7. Table  8. Bed9. Electric Mitad  10. Other (specify)_______ \| \| \| \| \| \|  \| \| \| 210 \| What is main current source of drinking water for members of your house hold? \| \| 1. Piped water  2. Public Tap/Stand Pipe  3. Borehole  4. Protected well  5. Unprotected well  6. Protected Spring  7. Unprotected Spring  8. River/Ponds/Stream/Dam  9. Other (specify) ______ \| \| \| \| \| \|  \| \| \| 211 \| What kind of toilet facility do members of your household usually use?  *(Record Observation)* \| \| 1. Pit latrine  2. Ventilated improved pit latrine  3. No facility/bush/field  4. Other (specify)_____ \| \| \| \| \| \|  \| \| \| 212 \| What are the main materials of the floor of house?  *(Record observation)* \| \| 1. Earth/Sand  2. Wood planks  3. Palm/Bamboo  4. Ceramic Tiles  5. Cement  6. Other (specify)______ \| \| \| \| \| \|  \| \| \| 213 \| What is main material of the exterior walls of house?  *(Record observation)* \| \| 1. Simple wall with mud or local materials  2. Bamboo or stone with mud, plywood, cardboard  3. Finished walls; cement, brick, stone with cement, wood planks  4. No outside walls  5. Others (specify)______ \| \| \| \| \| \|  \| \| \| 214 \| How many rooms are there for your house? \| \| ___________(in number) \| \| \| \| \| \|  \| \| \| 215 \| Do you have a separate room used as a kitchen? *(Record observation)* \| \| 1. Yes  2. No \| \| \| \| \| \|  \| \| \| 216 \| What type of fuel do you mainly use for cooking? \| \| 1. Electricity 2. Wood  3. Kerosene 4. Animal dung  5. Charcoal 6. Agricultural crops  7. Other (specify)_____ \| \| \| \| \| \|  \| \| \| 217 \| Does any member of this household have a bank or micro-finance saving account? \| \| 1. Yes  2. No \| \| \| \| \| \|  \| \| \| 218 \| How much of money? \| \| ___________(Ethiopian Birr \| \| \| \| \| \| \| \| \| **PART III: OBESTETRIC RELATED QUESIONS** \| \| \| \| \| \| \| \| \| \| \| \| 301 \| How many times you gate pregnant? \| \| ------------------ \| \| \| \| \| \|  \| \| \| 302 \| How many times you gave birth? \| \| ------------------ \| \| \| \| \| \|  \| \| \| 303 \| Of these pregnancies how many terminated before 7 months (28 weeks)? \| \|  \| \| \| \| \| \|  \| \| \| 304 \| Is the pregnancy wanted and planned? \| \| 1. Yes  2. No \| \| \| \| \| \|  \| \| \| 305 \| Do you have ANC visit during pregnancy? \| \| 1. Yes  2. No \| \| \| \| \| \| If “No” Skip to Q406 \| \| \| 306 \| How many times you received ANC service from health care provider?) \| \| ___________(in number \| \| \| \| \| \|  \| \| \| 307 \| Where did you give birth the last baby? \| \| 1. Health Center  2. Hospital  3. Health Post  4. Home \| \| \| \| \| \|  \| \| \| 308 \| Who attended/ assisted the delivery? \| \| 1. Health care provider  2. Family  3. Neighbor  4. mother in law  5. Traditional Birth Attendant  6. Health Extension Worker  7.Other, Specify_______________ \| \| \| \| \| \|  \| \| \| 309 \| In which mode of delivery you gave birth? \| \| 1. Spontaneous vaginal delivery  2. Instrumental assisted delivery  3. Caesarean section \| \| \| \| \| \| For institutional delivery only \| \| \| 310 \| Had you faced any type of complication during the delivery? \| \| 1. Yes  2. No \| \| \| \| \| \|  \| \| \| 311 \| What is the sex of baby \| \| 1. Male2. Female \| \| \| \| \| \|  \| \| \| 312 \| What was the desired sex of you \| \| 1. Male2. Female \| \| \| \| \| \|  \| \| \| 313 \| Does your child become ill? \| \| 1. Yes 2. No \| \| \| \| \| \|  \| \| \| 314 \| If yes, How the illness treated? \| \| 1. None  2. Outpatient  3. Admitted \| \| \| \| \| \|  \| \| \| 315 \| Do you have children loss in the previous delivery? \| \| 1. Yes  2. No \| \| \| \| \| \|  \| \| \| 316 \| Who gave care for you after delivery? \| \| 1. Health Extension Worker  2. Family/ mother in law  3. Neighbor  4. My mom  5.Other, Specify_______________ \| \| \| \| \| \|  \| \| \| **PART IV COVID- 19 related** \| \| \| \| \| \| \| \| \|  \| \| \| 401 \| You heard about covid 19? \| \| 1. Yes 2. No \| \| \| \| \| \|  \| \| \| 402 \| If yes have you tested? \| \| 1. Yes 2. No \| \| \| \| \| \|  \| \| \| 403 \| if yes for 402 what was the result? \| \| 1. Yes 2. No \| \| \| \| \| \|  \| \| \| 404 \| If yes for 401 have one of your family/ relative tested? \| \| 1. Yes 2. No \| \| \| \| \| \|  \| \| \| 405 \| Have you stayed lockdown at home at the time of the national state of the emergency announcement \| \| 1. Yes 2. No \| \| \| \| \| \|  \| \| \| 406 \| Have you apply recommended transmission prevention mechanisms? \| \| 1. Yes 2. No \| \| \| \| \| \|  \| \| \| 407 \| If yes for 406 what did you apply? \| \| 1. Social distancing 2. Physical distancing 3. Wearing mask 4. Hand washing 5. Stay at home 6. Use sanitizer \| \| \| \| \| \|  \| \| \| **PART V Decision Making** \| \| \| \| \| \| \| \| \|  \| \| \| 501 \| Who will decide on issues concerning with the house \| \| 1. Me 2. Husband/ co-habitant 3. Mother in low 4. My family 5. Other relatives \| \| \| \| \| \|  \| \| \| 502 \| Who will decide on your issues \| \| 1. Me 2. Husband/ co-habitant 3. Mother in low 4. My family 5. Other relatives \| \| \| \| \| \|  \| \| \| 503 \| Who was decided during marriage \| \| 1. Me 2. Husband/ co-habitant 3. Mother in low 4. My family 5. Other relatives \| \| \| \| \| \|  \| \| \| **PART VI Alcohol Use** \| \| \| \| \| \| \| \| \|  \| \| \| 601 \| Have you ever drunk alcohol in lifetime \| \| 1. Yes 2. No \| \| \| \| \| \|  \| \| \| 602 \| If yes for 601in what extent \| \| 1. Only once 2. During holy day 3. Yearly 4. Monthly 5. Weekly 6. Daily \| \| \| \| \| \|  \| \| \| 603 \| Have your husband/ co-habitant ever drunk alcohol in lifetime \| \| 1. Yes 2. No \| \| \| \| \| \|  \| \| \| 604 \| If yes for 603in what extent \| \| 1. Only once 2. During holy day 3. Yearly 4. Monthly 5. Weekly 6. Daily \| \| \| \| \| \|  \| \| \| 605 \| Have your husband/ co-habitant ever show one of the following? (Controlling behavior)  *(More than one answer is possible)* \| \| 1. Restricts you from seeing her friends, 2. Contact with your family 3. Insists on knowing where you all the time 4. Gets angry when you speaks with other men \| \| \| \| \| \|  \| \| \| **PART VII Intimate Partner Violence** \| \| \| \| \| \| \| \| \|  \| \| \| 701 \| Have your husband/ co-habitant ever show one of the following? (Physical violence)  *(More than one answer is possible)* \| \| 1. Something thrown to harm you 2. Pushed, hit with a fist or something else to hurt you 3. Kicked, drugged, burnet on purpose on you 4. Try to use or used gun, knife against you \| \| \| \| \| \|  \| \| \| 702 \| If yes for 701 when? \| \| 1. Before a year 2. Before covid-19 3. Less than one year 4. After covid-19 5. Other \| \| \| \| \| \|  \| \| \| 703 \| Have your husband/ co-habitant ever show one of the following? (Sexual violence)  *(More than one answer is possible)* \| \| 1. Forced sexual intercourse against your interest 2. Sexual intercourse when you didn't want to do 3. You forced to do something sexual that you perceives as humiliating \| \| \| \| \| \|  \| \| \| 704 \| If yes for 703 when? \| \| 1. Before a year 2. Before covid-19 3. Less than one year 4. After covid-19 5. Other \| \| \| \| \| \|  \| \| \| 705 \| Have your husband/ co-habitant ever show one of the following? (Emotional violence)  *(More than one answer is possible)* \| \| 1. You humiliated in front of others 2. You intimidated or scared on purpose 3. You ever threatened with harm \| \| \| \| \| \|  \| \| \| 706 \| If yes for 705 when? \| \| 1. Before a year 2. Before covid-19 3. Less than one year 4. After covid-19 5. Other \| \| \| \| \| \|  \| \| \| 707 \| What do you think about this \| \| 1. Something thrown to harm you   Good b. not good   1. Pushed, hit with a fist or something else to hurt you 2. Good b. not good 3. Kicked, drugged, burnet on purpose on you 4. Good b. not good 5. Try to use or used gun, knife against you 6. Good b. not good 7. Forced sexual intercourse against your interest 8. Good b. not good 9. Sexual intercourse when you didn't want to do 10. Good b. not good 11. You forced to do something sexual that you perceives as humiliating 12. Good b. not good 13. You humiliated in front of others 14. Good b. not good 15. You intimidated or scared on purpose 16. Good b. not good 17. You ever threatened with harm 18. Good b. not good \| \| \| \| \| \|  \| \| \| **PART VIII SOCIAL SUPPORT** \| \| \| \| \| \| \| \| \| \| \| \|  \|  \| \| \| 1.S. D \| \| 2. D \| 3.U \| 4. A \| \| 5.S.A \| \| 801 \| Whenever you need help, you ask your family for support? \| \|  \| \| \|  \|  \|  \| \|  \| \| 802 \| Do you agree your family’s friendship network is good? \| \|  \| \| \|  \|  \|  \| \|  \| \| 803 \| Do you agree your spouse assured you that you can rely completely on him? \| \|  \| \| \|  \|  \|  \| \|  \| \| 804 \| Do you have conflict with spouse in the past days? \| \|  \| \| \|  \|  \|  \| \|  \| \| 805 \| Do you agree you are feeling controlled by family? \| \|  \| \| \|  \|  \|  \| \|  \| \| 806 \| Do you agree your family showed you that they loves and accepts you? \| \|  \| \| \|  \|  \|  \| \|  \| |
| --- | --- | --- | --- | --- | --- | --- | --- | --- | --- | --- | --- | --- | --- | --- | --- | --- | --- | --- | --- | --- | --- | --- | --- | --- | --- | --- | --- | --- | --- | --- | --- | --- | --- | --- | --- | --- | --- | --- | --- | --- | --- | --- | --- | --- | --- | --- | --- | --- | --- | --- | --- | --- | --- | --- | --- | --- | --- | --- | --- | --- | --- | --- | --- | --- | --- | --- | --- | --- | --- | --- | --- | --- | --- | --- | --- | --- | --- | --- | --- | --- | --- | --- | --- | --- | --- | --- | --- | --- | --- | --- | --- | --- | --- | --- | --- | --- | --- | --- | --- | --- | --- | --- | --- | --- | --- | --- | --- | --- | --- | --- | --- | --- | --- | --- | --- | --- | --- | --- | --- | --- | --- | --- | --- | --- | --- | --- | --- | --- | --- | --- | --- | --- | --- | --- | --- | --- | --- | --- | --- | --- | --- | --- | --- | --- | --- | --- | --- | --- | --- | --- | --- | --- | --- | --- | --- | --- | --- | --- | --- | --- | --- | --- | --- | --- | --- | --- | --- | --- | --- | --- | --- | --- | --- | --- | --- | --- | --- | --- | --- | --- | --- | --- | --- | --- | --- | --- | --- | --- | --- | --- | --- | --- | --- | --- | --- | --- | --- | --- | --- | --- | --- | --- | --- | --- | --- | --- | --- | --- | --- | --- | --- | --- | --- | --- | --- | --- | --- | --- | --- | --- | --- | --- | --- | --- | --- | --- | --- | --- | --- | --- | --- | --- | --- | --- | --- | --- | --- | --- | --- | --- | --- | --- | --- | --- | --- | --- | --- | --- | --- | --- | --- | --- | --- | --- | --- | --- | --- | --- | --- | --- | --- | --- | --- | --- | --- | --- | --- | --- | --- | --- | --- | --- | --- | --- | --- | --- | --- | --- | --- | --- | --- | --- | --- | --- | --- | --- | --- | --- | --- | --- | --- | --- | --- | --- | --- | --- | --- | --- | --- | --- | --- | --- | --- | --- | --- | --- | --- | --- | --- | --- | --- | --- | --- | --- | --- | --- | --- | --- | --- | --- | --- | --- | --- | --- | --- | --- | --- | --- | --- | --- | --- | --- | --- | --- | --- | --- | --- | --- | --- | --- | --- | --- | --- | --- | --- | --- | --- | --- | --- | --- | --- | --- | --- | --- | --- | --- | --- | --- | --- | --- | --- | --- | --- | --- | --- | --- | --- | --- | --- | --- | --- | --- | --- | --- | --- | --- | --- | --- | --- | --- | --- | --- | --- | --- | --- | --- | --- | --- | --- | --- | --- | --- | --- | --- | --- | --- | --- | --- | --- | --- | --- | --- | --- | --- | --- | --- | --- | --- | --- | --- | --- | --- | --- | --- | --- | --- | --- | --- | --- | --- | --- | --- | --- | --- | --- | --- | --- | --- | --- | --- | --- | --- | --- | --- | --- | --- | --- | --- | --- | --- | --- | --- | --- | --- | --- | --- | --- | --- | --- | --- | --- | --- | --- | --- | --- | --- | --- | --- | --- | --- | --- | --- | --- | --- | --- | --- | --- | --- | --- | --- | --- | --- | --- | --- | --- | --- | --- | --- | --- | --- | --- | --- | --- | --- | --- | --- | --- | --- | --- | --- | --- | --- | --- | --- | --- | --- | --- | --- | --- | --- | --- | --- | --- | --- | --- | --- | --- | --- | --- | --- | --- | --- | --- | --- | --- | --- | --- | --- | --- | --- | --- | --- | --- | --- | --- | --- | --- | --- | --- | --- | --- | --- | --- | --- | --- | --- | --- | --- | --- | --- | --- | --- | --- | --- | --- | --- | --- | --- | --- | --- | --- | --- | --- | --- | --- | --- | --- | --- | --- | --- | --- | --- | --- | --- | --- | --- | --- | --- | --- | --- | --- | --- | --- | --- | --- | --- | --- | --- | --- | --- | --- | --- | --- | --- | --- | --- | --- | --- | --- | --- | --- | --- | --- | --- | --- | --- | --- | --- | --- | --- | --- | --- | --- | --- | --- | --- | --- | --- | --- | --- | --- | --- | --- | --- | --- | --- | --- | --- | --- | --- | --- | --- | --- | --- | --- | --- | --- | --- | --- | --- | --- | --- | --- | --- | --- | --- | --- | --- | --- | --- | --- | --- | --- | --- | --- | --- | --- | --- | --- | --- | --- | --- | --- | --- | --- | --- | --- | --- | --- | --- | --- | --- | --- | --- | --- | --- | --- | --- | --- | --- | --- | --- | --- | --- | --- | --- | --- | --- | --- | --- | --- | --- | --- | --- | --- | --- | --- | --- | --- | --- | --- | --- | --- | --- | --- | --- | --- | --- | --- | --- | --- | --- | --- | --- | --- | --- | --- | --- | --- | --- | --- | --- | --- | --- | --- | --- | --- | --- | --- | --- | --- | --- | --- | --- | --- | --- | --- | --- | --- | --- | --- | --- | --- | --- | --- | --- | --- | --- | --- | --- | --- | --- | --- | --- | --- | --- | --- | --- | --- | --- | --- | --- | --- | --- | --- | --- | --- | --- | --- | --- | --- | --- | --- | --- | --- | --- | --- | --- | --- | --- | --- | --- | --- | --- | --- | --- | --- | --- | --- | --- | --- | --- | --- | --- | --- | --- | --- | --- | --- | --- | --- | --- | --- | --- | --- | --- | --- | --- | --- | --- | --- | --- | --- | --- | --- | --- | --- | --- | --- | --- | --- | --- | --- | --- | --- | --- | --- | --- | --- | --- | --- | --- | --- | --- | --- | --- | --- | --- | --- | --- | --- | --- | --- | --- | --- | --- | --- | --- | --- | --- | --- | --- | --- | --- | --- | --- | --- | --- | --- | --- | --- | --- | --- | --- | --- | --- | --- | --- | --- | --- | --- | --- | --- | --- | --- | --- | --- | --- | --- | --- | --- | --- | --- | --- | --- | --- | --- | --- | --- | --- | --- | --- | --- | --- | --- | --- | --- | --- | --- | --- | --- | --- | --- | --- | --- |
|  |

**Thank You**
